# Nature-Inspired Antimicrobial Polymers – Assessment of Their Potential for Biomedical Applications

**DOI:** 10.1371/journal.pone.0073812

**Published:** 2013-09-09

**Authors:** Ali Al-Ahmad, Dougal Laird, Peng Zou, Pascal Tomakidi, Thorsten Steinberg, Karen Lienkamp

**Affiliations:** 1 Operative Dentistry and Periodontology, University Medical Center of the Albert-Ludwigs-Universität, Freiburg, Germany; 2 Freiburg Institute for Advanced Studies, Albert-Ludwigs-Universität, Freiburg, Germany; 3 Oral Biotechnology, University Medical Center of the Albert-Ludwigs-Universität, Freiburg, Germany; 4 Department of Microsystems Engineering, Albert-Ludwigs-Universität, Freiburg, Germany; Max Planck Institute for Polymer Research, Germany

## Abstract

We explored the potential of poly(oxonorbornene)-based synthetic mimics of antimicrobial peptides (SMAMPs), a promising new class of antimicrobial polymers with cell-selectivity and low resistance development potential, for clinical applications. We evaluated their antimicrobial activity against a panel of seven clinical and regulatory relevant bacteria strains, and tested their toxicity with two different kinds of primary human cells. For the antimicrobial activity, we performed the minimum inhibitory concentration (MIC) assay and determined the minimum bactericidal concentration (MBC) according to the NCCLS guidelines. The results revealed specific problems that may occur when testing the antimicrobial activity of amphiphilic cationic polymers, and confirmed the working hypothesis that the more hydrophilic SMAMP polymers in our portfolio were ‘doubly selective’, i.e. they are not only selective for bacteria over mammalian cells, but also for Gram-positive over Gram-negative bacteria. The data also showed that we could improve the broad-band activity of one SMAMP, and in combination with the results from the cell toxicity experiments, identified this polymer as a promising candidate for further in-vitro and in-vivo testing. Transmission electron studies revealed that the cellular envelopes of both *E. coli* and *S. aureus* were severely damaged due to SMAMP action on the bacterial membrane, which strengthened the argument that SMAMPs closely resemble antimicrobial peptides. To test cell toxicity, we used the traditional hemolysis assay with human red blood cells, and the novel xCelligence assay with primary human fibroblasts. The data reported here is the first example in which a hemolysis assay is benchmarked against the xCelligence assay. It revealed that the same trends were obtained using these complementary methods. This establishes the xCelligence assay with primary human cells as a useful tool for SMAMP characterization.

## Introduction

Hospital-acquired infections with bacteria cause severe healthcare problems, especially with immunocompromised patients. These infections may even be lethal for otherwise healthy individuals when they are caused by multi-resistant bacteria. Unfortunately, resistant strains are prevalent in healthcare facilities and public places in Europe and the USA.[1]–[3] While resistance rates in MRSA and *Enterococcus faecalis* currently remain static on a high level, the rates of resistant *Escherichia coli* and *Klebsiella pneumoniae* are still increasing. [4] Every year, 25 000 people in Europe die from infections with multi-resistant bacteria. [4] Besides the human suffering, this adds an estimated 900 million Euro per year to the European states’ healthcare budget. [4] Thus, new and efficient antimicrobial substances constitute an immediate need and, in addition to strict hygiene protocols, can help contain hospital-acquired bacterial infections. It is of particular importance that such substances should show significantly lower resistance formation potential than common antibiotics, to avoid yet another vicious circle of resistance formation.

**Figure 1:**
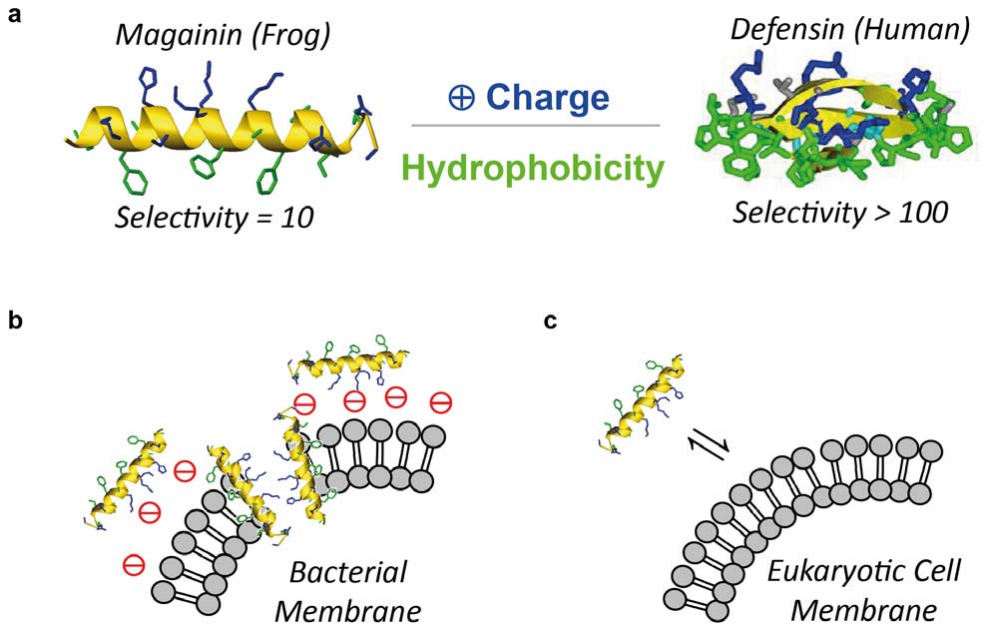
Cartoon illustrations. a) Facial amphiphilicity of the antimicrobial peptides magainin and defensin. The peptide backbone (yellow) directs the hydrophilic, cationic groups (blue) to one face of the molecule, while the hydrophobic groups (green) segregate to the opposite face of the molecule. b) Interaction of AMPs with bacterial plasma membranes. Details, such as bacterial lipopolysaccharides (Gram-negative bacteria) or peptidoglycan layers (Gram-positive and Gram-bacteria), or the precise pre-arrangement of AMPs before insertion through the membrane, are omitted for reasons of clarity; c) Interaction of AMPs with charge neutral mammalian cells. Due to the lack of net negative charge on the cell membrane, the interaction is reversible, which reduces the AMP’s propensity to insert through the membrane.

One of nature’s solutions to fend off bacterial intruders are antimicrobial peptides (AMPs). These host-defense peptides have secondary structures that direct their polar, cationic residues to one side of the molecule, while their hydrophobic residues segregate to the opposite side (Fig. 1a). The resulting facial amphiphilicity enables AMPs to attach to negatively charged bacterial membranes with the hydrophilic face, and then insert through that membrane with the hydrophobic face (Fig. 1b). [5], [6] Mammalian cells, on the other hand, are overall charge neutral, which is why AMPs experience no electrostatic attraction to these cells’ plasma membrane (Fig 1c). [5], [6] As a result, AMPs are compatible with the body cells of their parent organism, but active against bacterial pathogens. [5], [6] This is referred to as cell selectivity, and is typically quantified by taking the ratio of the AMP’s ability to lyse human erythrocytes ( = HC_50_ value, see below), and the AMP’s antimicrobial activity ( = MIC_90_, see below). Based on these parameters, the frog AMP magainin has a selectivity of 10, that of human AMPs (defensins) is >100. As the AMP-bacteria interaction is unspecific compared to the very specific cell targets of antibiotics, bacteria are less prone to develop resistance to AMPs. While AMPs have been around for hundreds of thousands of years, the amount of AMP resistance observed is negligible compared to the high resistance rates in clinically used antibiotics. [5], [11].

We and others recently showed that we can teach synthetic polymers to behave like AMPs. This was achieved by carefully designing the distribution of the chemical functional groups on the polymer backbone, so that the polymers were also facially amphiphilic.[7]–[13] It was further demonstrated that such polymer-based synthetic mimics of antimicrobial peptides (SMAMPs) also target the bacterial membrane, most likely by a mechanism similar to AMPs. [8], [14], [15] Conventional antimicrobial polymers, which have already been reported decades ago, are usually biocidal - they cannot differentiate between bacteria and host cells. Polymer SMAMPs, on the other hand, have been shown to be as cell selective as AMPs. [7], [10] It was also demonstrated that the potential of SMAMPs to cause bacterial resistance is low. For example, *E. coli* bacteria which had been 21 times exposed to sublethal doses of poly(methacrylate)-based SMAMPs did not develop resistance, while the minimum inhibitory concentration of the antibiotics Ciprofloxacin and Norfloxacin against these bacteria increased by a factor of 250–500 in the same experimental set-up. [16] The limiting factor that so far prevented bringing AMPs into applications was not only their propensity to undergo enzymatic degeneration, but also their limited availability. SMAMPs do not have these drawbacks. Thus, the combined properties of excellent antimicrobial activity, cell selectivity, low resistance formation potential and easy availability make SMAMPs ideal candidates for biomedical applications.

**Figure 2:**
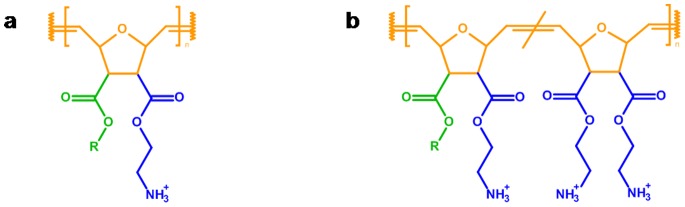
Chemical structure of poly(oxonorbornene)-based synthetic mimics of antimicrobial peptides (SMAMPs). a) SMAMPs with tunable antimicrobial activity and cell-compatibility can be obtained by varying the hydrophobic groups (green, R = methyl to hexyl) and hydrophilic groups (blue) that are attached to the poly(oxonorbornene backbone) (orange). b) Structure of the SMAMP copolymers used in this study; Series 1: R = Propyl, Series 2: R = Butyl.

We have recently developed a facile synthetic platform (‘molecular construction kit’) to obtain poly(oxonorbornene)-based SMAMPs (Fig. 2a). [7] These SMAMPs had excellent AMP-like antimicrobial properties, which could be tuned over two orders of magnitude. [7] The advantage of poly(oxonorbornene) SMAMPs over other polymer families is their precisely defined local amphiphilicity on the repeat unit level, which closely mimics the AMP structure and allows accurate fine tuning of the antimicrobial activity and cell selectivity. [7] Additionally, the molecular structure of their backbone allows further chemical functionalization, for example covalent attachment to a surface, and intermolecular cross-linking. [17] This is relevant for the fabrication of biomedical materials such as polymer-coated catheters or implants.

The focus of our previous studies on poly(oxonorbornene) SMAMPs was on discovering and understanding the structural parameters that control antimicrobial activity and biocompatibility, which we tested by determining the minimum inhibitory concentration (MIC) of the SMAMPs against laboratory strains of *E. coli* and *S. aureus*, and the SMAMP concentration that caused 50% hemolysis in red blood cells (HC_50_).[7]–[9] In this study, we explore the potential of these SMAMPs for clinical applications. We had two aims in mind. First, as our most cell-selective SMAMPs were so far only active against Gram-negative strains, [7], [8] we wanted to synthesize SMAMPs with improved antimicrobial broad-band activity. Secondly, to be able to assess their potential for biomedical applications, we wanted to know more about their antimicrobial activity and biocompatibility on the in-vitro level. To that end, we tested their antimicrobial activity against a panel of seven clinical and regulatory relevant bacteria strains, and compared the resulting data from two antimicrobial assays - the minimum inhibitory concentration (MIC) assay and the minimum bactericidal concentration (MBC) according to the NCCLS guidelines (now: Clinical and Laboratory Standards Institute, CLSI). [18], [19] This revealed specific problems that may occur when testing amphiphilic cationic polymers. We also used two complementary methods to assess cell compatibility – the traditional hemolysis assay with human red blood cells yielding the HC_50_, and the xCelligence assay, a relatively novel approach that monitors the viability of cells in real time using impedance spectroscopy.[20]–[22] We performed the latter assay with primary human fibroblast cells. The rationale for this was that primary human cells are a much better model system for healthy human physiology than rodent cell lines, immortalized non-tissue specific human cell lines, and even in-vivo rodent models.[23]–[27] The resulting data gave us deeper insights into the antimicrobial activity and cell compatibility of our SMAMPs, and thus allowed a better assessment of their potential for biomedical applications. In particular, one SMAMP polymer was identified as a promising candidate for further in-vitro and in-vivo testing. Additionally, the cell compatibility data here reported, to our knowledge, is the first example in which a hemolysis assay is benchmarked against the xCelligence assay. It reveals that the same trends are obtained using these complementary methods.

## Results and Discussion

### Synthesis

In our search for broad-band active poly(oxonorbornene) SMAMPs, we took the homopolymer with R = propyl (Fig. 2a) as the starting point. This polymer was previously shown to be active against Gram-negative *Escherichia coli* and Gram-positive *Staphylococcus aureus*, yet it was strongly hemolytic and therefore not cell selective. [7] As cell compatibility in AMPs and SMAMPs is generally improved by increasing the overall polymer hydrophilicity, [28] we added the more hydrophilic, two-fold positively charged diamine repeat units to that structure (Fig. 2b). We synthesized two series of copolymers for this study, one based on the propyl SMAMP shown in Fig. 2a (Series 1, Fig. 2b), the other based on its more hydrophobic butyl homologue (Series 2, Fig. 2b). Details of the polymer synthesis and physical characterization are given in the supporting information (Figure S1: synthesis scheme, Table S1: experimental parameters, Table S2: polymer characterization results, Figure S2: GPC elugrams, Figure S3 and S4: NMR spectra of precursors and active polymers). In short, we copolymerized the amphiphilic propyl- or butyl-oxonorbornene monomer and the diamine-oxonorbornene monomer (all of them with a protective group on the amines) via ring-opening metathesis polymerization, using Grubbs’ catalyst as an initiator. The repeat unit ratio of the amphiphilic repeat unit (propyl ( = P) or butyl ( = B)) and the hydrophilic repeat unit ( = D) was varied from 0% to 90% D. The abbreviations describing each sample were chosen accordingly. For example, P:D = 5∶5 denotes a copolymer with 50% propyl and 50% diamine repeat units. Compared to previous reports which did not give the desired broad-band activity, [9] we varied the ratio of the hydrophilic to amphiphilic repeat unit in both series over a wider range, and targeted lower molecular weights (3000 g/mol instead of 5000 g/mol). This corresponds to the molecular weight range and number of repeat units of typical AMPs, and was the molecular weight range that yielded the most active SMAMPs in previous studies.[7]–[9].

### Antimicrobial Activity

**Figure 3:**
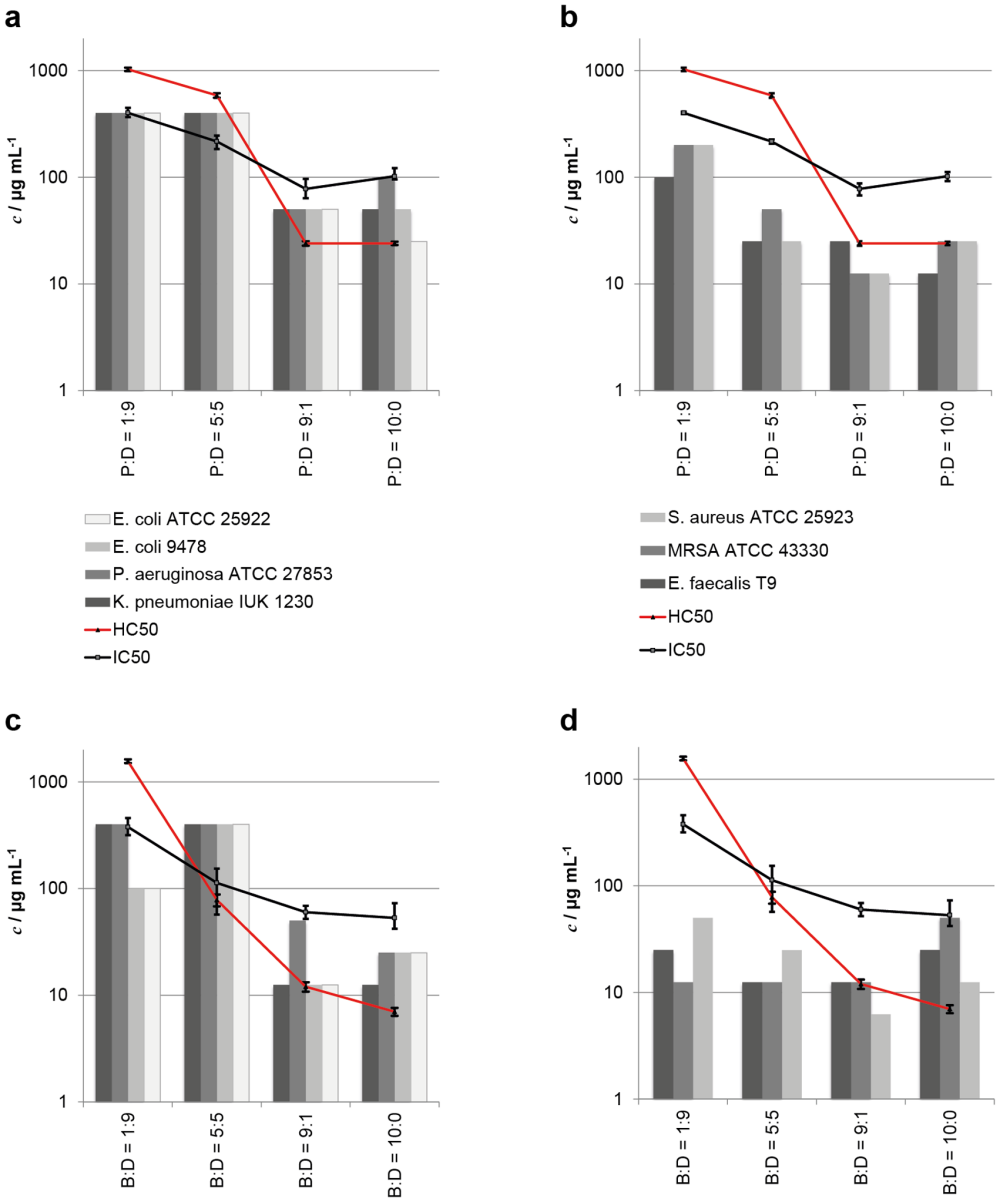
Antimicrobial activity (MIC_90_ or MIC (optical), respectively) and cell compatibility (HC_50_ and IC_50_) of the SMAMP series as function of increasing polymer hydrophobicity. a) Series 1, Gram-negative bacteria, b) Series 1, Gram-positive bacteria, c) Series 2, Gram-negative bacteria, d) Series 2, Gram-positive bacteria.

To evaluate the antimicrobial activity of the two polymer series, we tested their minimum inhibitory concentration (MIC) against the Gram-negative strains *E. coli* ATCC 25922, *E. coli* 9478, *Pseudomonas aeruginosa* ATCC 27853, *Klebsiella pneumoniae* IUK 1230, and the Gram-positive strains *S. aureus* ATCC 25923, MRSA ATCC 43330 and *Enteriococcus faecalis* T9. The rationale behind choosing these bacteria was that they are relevant in many infectious diseases (e.g. pneumonia, meningitis, bacteremia, osteomyelitis, endocarditis, toxic shock syndrome, gastroenteritis, urinary tract infection, and sepsis), and that they are of regulatory relevance (European pharmacopeia). In particular, we tested MRSA as an example of a pathogen that causes severe medical device-related infections, and *E. faecalis* T9 as an example of strongly biofilm forming bacteria. Details of the MIC assay are given in Text S1. The results of all antimicrobial tests are shown in Tables 1 and 2, and in Fig. 3. In this figure, the polymers of Series 1 (Fig. 3a and 3b) and Series 2 (Fig. 3c and 3d) were arranged by increasing hydrophobicity along the x-axis. The antimicrobial activity is plotted separately for the Gram-negative and the Gram-positive bacteria. The optical MIC, i.e. the concentration at which no bacterial growth is observed visually, is reported in Tables 1 and 2. We also quantified the bacterial growth as a function of SMAMP concentration via optical density (OD) measurements using a plate reader. The MIC_90_ can thus be defined as the SMAMP concentration at which 90% bacterial growth is inhibited according to the OD measurement (Tables 1 and 2). This OD vs. concentration data usually yields a sigmoidal curve, which we also observed for the more hydrophilic polymers within each series. However, the curves for the hydrophobic SMAMPs all went through a minimum. In some cases, this minimum was observed at 5% growth, which then still allowed defining the MIC_90_. In other cases, the growth at the curve minimum was much larger than 10%, which meant that an MIC_90_ could not be defined. This turned out, in some cases, to be a normalization artifact (a high growth percentage was calculated even though the solution was transparent to the eye). In other cases, significant turbidity of the solution was observed. This is not necessarily due to bacterial growth, but may also be a precipitated polyelectrolyte complex consisting of the inoculated dead bacteria and the cationic, more hydrophobic SMAMPs. To differentiate the two cases, we also plated out the respective samples on an agar plate and determined the minimal bactericidal concentration (MBC, Tables 1 and 2), which is the concentration where a three log reduction of bacterial growth ( = 99.9%) compared to a control is observed. As can be seen by comparing MIC (optical), MIC_90_ and MBC (Tables 1 and 2), those data points are in good numerical agreement for the hydrophilic samples. For the more hydrophobic samples, where no MIC_90_ could be defined, the optically determined MIC was usually equal to the MBC. Overall, this procedure allowed assessing the antimicrobial activity of our hydrophobic polymers without the bias of the OD-related measurement or calculation artifacts. In Fig. 3, the MIC_90_ was plotted whenever it was available, otherwise the MIC (optical) was used. The data in Fig. 3 shows that an increase in hydrophobicity increases the antimicrobial activity, while the hemolysis is simultaneously enhanced. This was anticipated. [7] The more hydrophilic members of each series (50% and 90% propyl or butyl content, respectively) were much more active against all Gram-positive bacteria tested, than against the Gram-negative bacteria, sometimes by a factor of 30. This ‘double selectivity’ had been previously assumed after testing laboratory strains of *E. coli* and *S. aureus* only. [9] With our larger test panel of bacteria, the Gram-selectivity hypothesis can now be confirmed. Additionally, an improved broad-band activity against all bacteria in our panel was observed for the more hydrophobic SMAMPs (P:D = 10∶0, P:D = 9∶1, B:D = 10∶0 and B:D = 9∶1, with a MIC_90_ of 12.5 to 50 µg/mL). Also, the hydrophilic B:D = 1∶9 was found to be reasonably active against all bacteria except *K. pneumoniae* and *P. aeruginosa.* The consequences of these findings are discussed below.

**Table 1:** Antibacterial activity in µg mL^−1^ of the SMAMP polymers (Series 1).

Sample	P:D = 10∶0				P:D = 9∶1				P:D = 5∶5				P:D = 1∶9			
c/µg mL^−1^	MIC (optical)	MBC	MIC OD	MIC90	MIC (optical)	MBC	MIC OD	MIC90	MIC (optical)	MBC	MIC OD	MIC90	MIC (optical)	MBC	MIC OD	MIC90
*S. aureus* ATCC 25923	25	25	25 (15)	nd	12.5	12.5	12.5 (13)	nd	25	50	25 (6)	25	200	200	200 (57)	nd
*E. coli* ATCC 25922	25	25	25 (4)	25	50	50	50 (3)	50	>400	>400	>400	>400	>400	>400	>400	>400
*E. coli* 9478	50	50	50 (7)	50	50	50	50 (4)	50	>400	>400	>400	>400	>400	>400	>400	>400
*Enterococcus faecalis* T9	12.5	12.5	12.5 (12)	nd	25	100	25 (13)	nd	25	50	25 (5)	25	100	100	100 (18)	nd
MRSA ATCC 43330	12.5	12.5	25 (4)	25	12.5	12.5	25 (5)	25	50	50	50 (4)	50	200	400	200 (12)	nd
*Pseudomonas aeruginosa* ATCC 27853	100	100	100 (6)	100	50	50	50 (4)	50	>400	>400	>400	>400	>400	>400	>400	>400
*Klebsiella pneumoniae* IUK 1230	50	50	50 (4)	50	50	50	50 (4)	50	>400	>400	>400	>400	>400	>400	>400	>400

MIC optical = concentration at which no bacterial growth was visible, MIC OD = concentration at which the optical density measurement indicated minimal bacterial growth (calculated percentages of bacterial growth in brackets), MIC_90_ =  concentration at which the bacterial growth was below 10% according to the optical density measurement, MBC = concentration at which the number of colony forming units was reduced by 99.9%; nd = not definable, blank cell = not determined.

**Table 2:** Antibacterial activity in µg mL^−1^ of the SMAMP polymers (Series 2).

Sample	B:D = 10∶0				B:D = 9∶1				B:D = 5∶5				B:D = 1∶9			
c/µg mL^−1^	MIC (optical)	MBC	MIC OD	MIC90	MIC (optical)	MBC	MIC OD	MIC90	MIC (optical)	MBC	MIC OD	MIC90	MIC (optical)	MBC	MIC OD	MIC90
*S. aureus* ATCC25923	12.5	12.5	12.5 (38)	nd	6.3	25	6.25 (29)	n.d.	25	50	25 (8)	25	50	200	50 (8)	50
*E. coli* ATCC25922	25	25	25 (12)	nd	12.5	25	12.5 (5)	12.5	>400	>400	>400	>400	100	n.b.	100 (5)	100
*E. coli* 9478	25	25	25 (11)	nd	12.5	50	12.5 (9)	12.5	>400	>400	>400	>400	100	n.b.	100 (4)	100
*Enterococcus* *faecalis* T9	25		25 (13)	nd	12.5	50	12.5 (33)	n.d.	12.5	200	12.5 (5)	12.5	25	200	25 (7)	25
MRSA ATCC43330	50	50	50 (5)	50	12.5	12.5	12.5 (5)	12.5	12.5	12.5	12.5 (10)	12.5	12.5	25	12.5 (12)	n.d.
*Pseudomonas* *aeruginosa*ATCC 27853	25		25 (18)	nd	50	100	50 (9)	50	>400	>400	>400	>400	400		400 (7)	400
*Klebsiella* *pneumoniae*IUK 1230	12.5		12.5 (6)	12.5	12.5	12.5	12.5 (4)	12.5	>400	>400	>400	>400	400		400 (5)	400

MIC optical = concentration at which no bacterial growth was visible, MIC OD = concentration at which the optical density measurement indicated minimal bacterial growth (calculated percentages of bacterial growth in brackets), MIC_90_ =  concentration at which the bacterial growth was below 10% according to the optical density measurement, MBC = concentration at which the number of colony forming units was reduced by 99.9%; nd = not definable, blank cell = not determined.

### Transmission Electron Microscopy (TEM)

**Figure 4:**
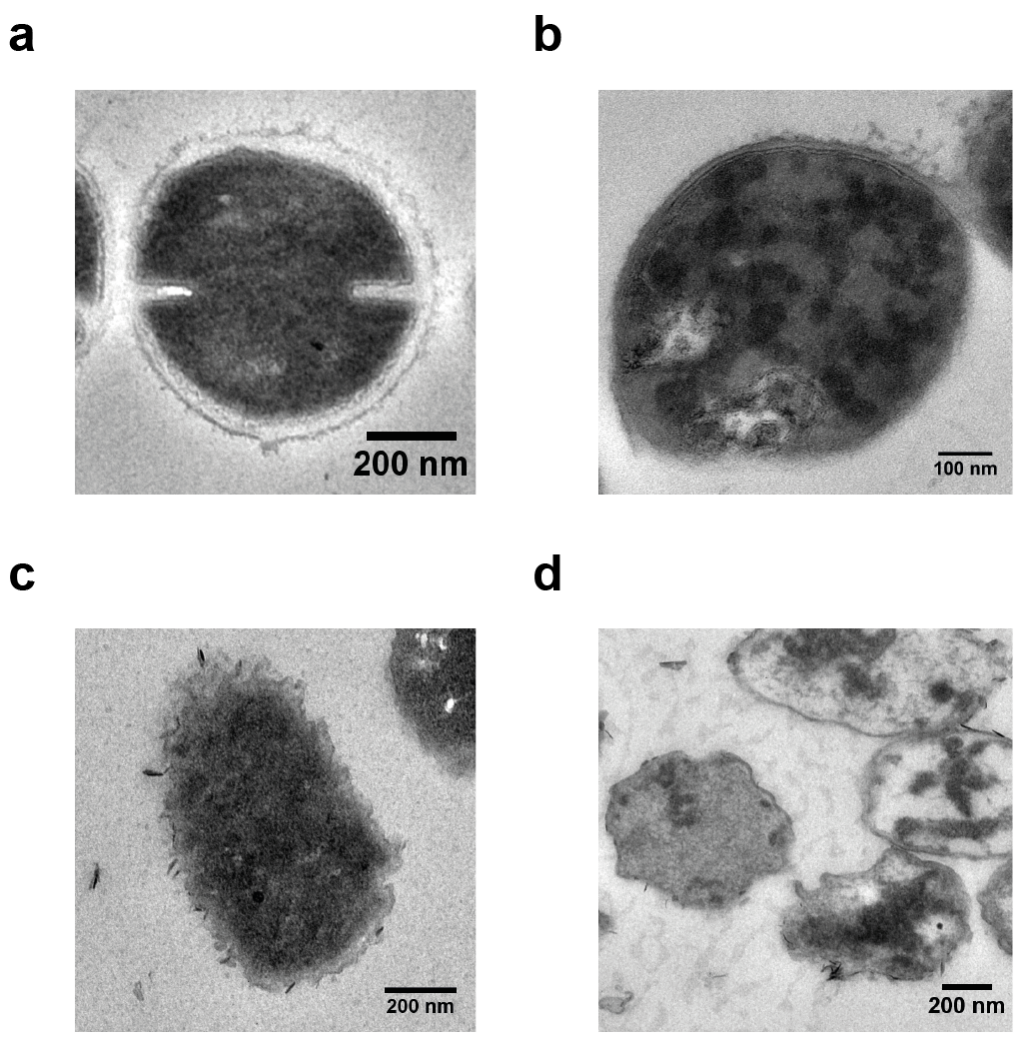
Transmission electron micrographs of *E. coli* and *S. aureus* bacteria that were stained, embedded in a Durcopan matrix, and microtomed as described in the supporting information. a) Untreated *S. aureus*, b) *S. aureus* incubated with SMAMP (P:D = 10∶0, 100 µg mL^−1^, 4 hours, at 37°C), c) Untreated *E. coli*, d) *E. coli* incubated with SMAMP (P:D = 10∶0, 100 µg mL^−1^, 4 hours, at 37°C).

Numerous studies (dye-leakage experiments on model vesicles,[14], [29]–[33] live-dead-staining of bacteria combined with fluorescence microscopy, [14], [31] etc.) have been conducted to elucidate the mechanism of SMAMP interaction with bacteria. Transmission electron microscopy studies, however, remain scarce. [34] We incubated *E. coli* and *S. aureus* bacteria for 4 hours with SMAMP P:D = 10∶0 at or above the MIC_90_. The cells were then embedded into a matrix, microtomed, and contrasted. Details of the sample preparation are given in Text S1. The resulting TEM images are shown in Fig. 4. The cells of the untreated controls (Fig. 4a and c) revealed dense structures and continuous cell envelopes. Treated *S. aureus* cells (Fig. 4b) showed a fuzzy peptidoglycan layer, discontinuous plasma membranes, and a less dense cell structure. Additionally, while most *S. aureus* bacteria in the untreated control were in the process of dividing, none of the treated *S. aureus* bacteria was. All this is indicative of damaged cell envelopes and a lethal efflux of the cell contents. The effect on *E. coli* bacteria was even more dramatic (Fig. 4d). The treated bacteria had numerous concave notches and showed significant loss of cell contents, with only cell ghosts and disintegrated cell envelopes remaining. The only other publication known to us that studied SMAMP polymers with TEM came to qualitatively similar conclusions, however less cell damage was observed there due to less active polymers. [34] Our results thus strengthen the currently accepted theory, i.e. that SMAMP-bacteria interactions are similar to AMP-bacteria interactions. The data does not reveal, however, what the exact mechanism of that interaction is.

### Cell Compatibility

As a simple screening parameter for cell compatibility, we performed the well-established hemolysis assay with our SMAMPs. In this assay, the leakage of hemoglobin from human red blood cells (RBCs) is monitored as a function of SMAMP concentration (Figure S5 in the supporting information). The concentration that led to 50% hemolysis ( =  HC_50_) was determined, and has been included in Fig. 3. Details of the hemolysis assay are given in Text S1. For both SMAMP series, the anticipated trend of increased hemolytic activity ( =  decrease in HC_50_) with increased polymer hydrophobicity was observed.

**Figure 5:**
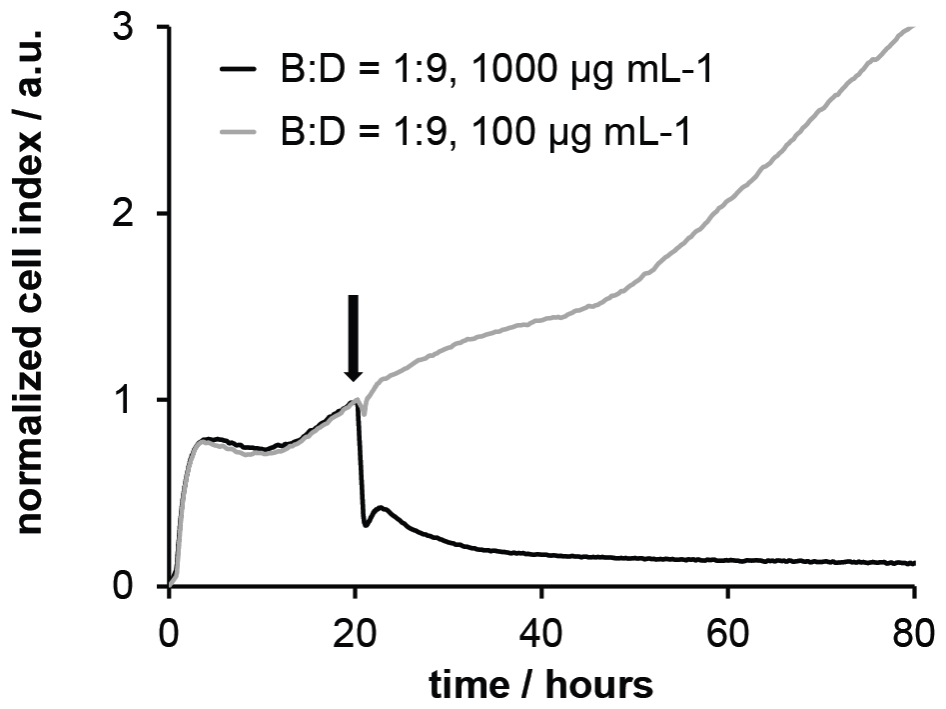
Cell index vs. time plot of a typical xCelligence experiment. Cells were seeded at t = 0 h; the test substance was added about t = 20 h (arrow in the figure). At low SMAMP concentration (grey curve), the change in the impedance signal continues to increase after SMAMP addition, indicating cell proliferation. At high SMAMP concentration, the signal drops, indicating cell detachment from the plate, and thus toxicity.

As a further measure for cell viability, the xCelligence assay (Roche) was used.[20]–[22] This assay is based on impedance spectroscopy. Cells are seeded into 16-well E-plates (Roche) with built-in gold micro-electrodes at the bottom of each well. When exposed to an alternating voltage, the electrical impedance in each well is measured simultaneously. The growing cells or cell sheets on the bottom electrode essentially behave like a dielectric layer with time-dependent impedance. Changes in the electrical impedance signal (called the Cell Index, CI) are influenced by the changes within the cell layer, from which we can infer processes such as cell proliferation, apoptosis, or cell detachment from the substrate in real-time. [35] The standard xCelligence experiment has three phases. First, cells are seeded on the substrate. They attach to the surface, which naturally increases the impedance, resulting in linear CI increase until a plateau is reached, at which point surface attachment is complete. After this plateau, cell proliferation occurs, upon which the CI increases further linearly. Undisturbed samples would continue to show this linear growth. Thus, when a test substance is added, changes in this part of the curve can be interpreted as continued proliferation (CI increase continues) or compromised viability due to cytotoxicity (CI signal is reduced). [36] It has been previously demonstrated that the CI is proportional to the cell number on the well surface under defined physiological conditions, while changes in the physiological conditions, e.g. adding a toxic test substance, may cause morphological changes in the cells (swelling, contraction, surface detachment), which naturally alters their dielectric properties and thus the CI signal. [22], [36], [37] By varying the concentration of the test substance, and plotting the CI vs. concentration, the substance amount at which cell viability is compromised by 50% (IC_50_) can be determined (Figure S6, S7 and S8 in the Supporting Information). IC_50_ data has been previously benchmarked against standard toxicity assays such as the XTT end point assay, [36] and the results were found to be in good agreement both qualitatively and quantitatively. [36] The advantage of the xCelligence assay is that it is label free and thus represents a chemically undisturbed test system. Further details are given in Text S1. In the xCelligence protocol used by us, the CI signal change in primary human gingiva fibroblasts upon exposure to SMAMPs (concentration range 10 to 2000 µg/mL) was measured. Typical CI signals for non-toxic as well as toxic SMAMP dilutions are shown in Fig. 5. As can be seen from this data, the typical CI behavior for toxic and non-toxic agents is also observed at different SMAMP concentration, indicating that the assay works well for our system. The resulting IC_50_ values for each polymer are included in Fig. 3. When comparing the HC_50_ data from the hemolysis assay and the IC_50_ data from the xCelligence assay, the same trends are obtained for each SMAMP series. However, the numerical results differ by as much as an order of magnitude. Since benchmarking of xCelligence data with other cell viability assays (performed on the same cell type) gave also numerical agreement of the respective data, [36] the reason of numerical differences between the hemolysis and xCelligence is attributed to the fundamental difference between human erythrocytes and the more complex primary human fibroblasts. Indeed, the hemolysis assay is good screening tool to assess cell compatibility; however erythrocytes are also very sensitive. As they have no metabolism, there is no cellular repair mechanism. As a result, their response to a test substance may over-amplify the actual toxicity of that substance. On the other hand, more complex primary cells may suffer from toxic pathways other than mere membrane damage that is not visible in erythrocytes. It is thus very plausible that assays with cells capable of proliferation, immune response and cell repair give different numerical results compared to the simple hemolysis assay. Still, it is extremely encouraging that the same trends are observed in both assays. This demonstrates that the xCelligence assay is a suitable method to study the effect of SMAMPs on cell viability. Since this assay, with primary human cells, is also a closer physiological model than experiments using rodent cells or even immortalized human cell lines, we are confident that our IC_50_ data also is a good guideline for the SMAMP concentration range that is safe for in-vivo testing.

We have so far only discussed trends in the MIC or the cell viability data. For any application purposes, however, the cell selectivity of our SMAMPs is relevant. As we have now two cell viability parameters, we can calculate a HC_50_/MIC selectivity, as well as the IC_50_/MIC selectivity. The results are shown in Table 3. Due to the numerical differences between HC_50_ and IC_50_, of course also different numerical selectivity data were obtained. When looking at these two sets of selectivity data, the following is observed for our SMAMPs: Overall, a ‘blockbuster’ polymer was not found with this experimental design. However, with HC_50_/MIC_90_ selectivities of 125 against MRSA, 63 against *E. faecalis* and 32 against *S. aureus*, the SMAMP B:D = 1∶9 may be a promising candidate to fight infections caused by Gram-positive bacteria. The HC_50_/MIC_90_ selectivities against Gram-negative *E. coli* are lower, yet still reasonable. However the polymer is not fully broad-band active, as the HC_50_/MIC_90_ selectivities for *P. aeruginosa* and *K. pneumoniae* are only 4. The same trends are also observed when looking at the IC_50_/MIC_90_ selectivities. Both the HC_50_/MIC_90_ and the IC_50_/MIC_90_ selectivities can be considered as an in-vitro analogue of the therapeutic index TI (defined as TD_50_/ED_50_, that is the ratio of the dose that causes toxic effects in 50% of patients over the minimal effective dose for 50% patients). Values for the TI of clinically used drugs range from the extremely benign penicillin (TI about 1000) to a TI of about 5 for paracetamol. In comparison to this data, we would identify B:D = 1∶9 as the only polymer in this portfolio that we would recommend for further in-vitro and in-vivo testing, at least for drug applications.

**Table 3:** Cell selectivity of the SMAMP polymers expressed in terms of HC_50_/MIC_90_ selectivity and IC_50_/MIC_90_ selectivity.

	Selectivity = HC_50_/MIC_90_								Selectivity = IC_50_/MIC_90_							
Sample	P:D = 10∶0	P:D = 9∶1	P:D = 5∶5	P:D = 1∶9	B:D = 10∶0	B:D = 9∶1	B:D = 5∶5	B:D = 1∶9	P:D = 10∶0	P:D = 9∶1	P:D = 5∶5	P:D = 1∶9	B:D = 10∶0	B:D = 9∶1	B:D = 5∶5	B:D = 1∶9
*S. aureus* ATCC 25923	1	2	23	5	1	2	3	31	4	6	9	2	4	10	5	8
*E. coli* ATCC 25922	1	0	1	3	0	1	0	16	4	2	1	1	2	5	0	4
*E. coli* 9478	0	0	1	3	0	1	0	16	2	2	1	1	2	5	0	4
*Enterococcus faecalis* T9	2	1	23	10	0	1	6	63	8	3	9	4	2	5	9	15
MRSA ATCC 43330	1	2	12	5	0	1	6	125	4	6	4	2	1	5	9	30
*Pseudomonas aeruginosa* ATCC 27853	0	0	1	3	0	0	0	4	1	2	1	1	2	1	0	1
*Klebsiella pneumoniae* IUK 1230	0	0	1	3	1	1	0	4	2	2	1	1	4	5	0	1
IC_50_	102	78	217	402	53	60	113	378								
HC_50_	24	24	585	1028	7	12	78	1566								

All data was calculated using the MIC_90_ values reported in Tables 1 and 2, or the MBC if MIC_90_ was unavailable.

## Conclusion

We have evaluated two series of antibacterial, cell-selective SMAMP polymers for their biomedical application potential. This involved testing each of our eight polymers against 7 bacterial strains using two different antimicrobial assays, and using the hemolysis assay and the xCelligence assay as two complementary methods to test the compatibility of these SMAMP polymers with primary human cells. The results with our broader test panel of bacteria confirmed that the more hydrophilic SMAMP polymers in our portfolio are ‘doubly selective’, i.e. they are not only selective for bacteria over mammalian cells, but also for Gram-positive over Gram-negative bacteria. This had so far only been a working hypothesis based on results for a single Gram-negative and Gram-positve bacterial strain. We could also improve the broad-band activity of B:D = 1∶9, and in combination with the results from the cell compatibility tests, identified this polymer as a promising candidate for further in-vitro and in-vivo testing. Transmission electron studies on one of our polymers further revealed that the cellular envelopes of both *E. coli* and *S. aureus* are severely damaged due to SMAMP action on the bacterial membrane. This further strengthens the argument that SMAMP action closely resembles AMP action. Finally, the cell compatibility assays (hemolysis and xCelligence), which are here benchmarked for the first time, revealed the same trends for both polymer series. We thus conclude that the xCelligence assay is a useful tool for SMAMP characterization, as primary human fibroblasts are a better model system to predict in-vivo performance of our SMAMPs than simple erythrocytes. However, more data on various other SMAMP and AMP systems is needed to get a better feeling for the numerical output of this assay.

When thinking about biomedical applications beyond mere drugs, e.g. antimicrobial coatings for catheters or implants, one has to bear in mind that the mechanism of antimicrobial surface activity and in-solution activity of polymers is different, even for one and the same polymer. Our next study will therefore involve the surface immobilization of the here reported SMAMP polymers, and the evaluation of their antimicrobial activity and biocompatibility on surfaces. We are currently working on this project, and will report our results in due course.

## Supporting Information

Figure S1 **Copolymer synthesis.** The monomers were obtained by ring-opening of oxonorbornene anhydride with the respective alcohol. The unreacted acid group was then further esterified. The monomers were mixed in the appropriate ratio (Table S1) and polymerized using Grubbs 3^rd^ generation catalyst. After quenching the living polymerization with ethylvinyl ether, deprotection with trifluoroacetic acid yielded the desired SMAMP copolymers.
(TIF)

Figure S2 **Overlay of GPC elugrams (refractive index detector signal (in arbitrary units) vs. elution time) of the precursor polymers (suffix –P).** a) propyl-containing polymers (Series 1), b) butyl-containing polymers (Series 2).
(TIF)

Figure S3 **^1^H-NMR spectra (250 MHz, CDCl_3_) of precursor polymers (suffix –P).** a) propyl-containing polymers (Series 1), b) butyl-containing polymers (Series 2).
(TIF)

Figure S4 **^1^H-NMR spectra (250 MHz, CDCl_3_) of the SMAMP polymers.** a) propyl-containing polymers (Series 1), b) butyl-containing polymers (Series 2).
(TIF)

Figure S5 **Results of the hemolysis assay.** The percentage of hemolysis is plotted vs. log_10_ of SMAMP concentration, yielding the HC_50_ at the point of inflection; a) Series 1, b) Series 2.
(TIF)

Figure S6 **Sigmoidal curve fitted to the concentration dependent effect of DMSO on the proliferation of gingiva fibroblast cells over 72 hours (IC_50_ = 0.86 v/v %).**
(TIF)

Figure S7 **xCelligence plot showing the effect of SMAMP B:D = 1∶9 at various concentrations on gingiva fibroblast cell proliferation.**
(TIF)

Figure S8 **Results of the xCelligence assay.** The normalized area under the curve is plotted vs. log_10_ of SMAMP concentration, yielding the IC_50_ at the point of inflection; a) Series 1, b) Series 2.
(TIF)

Table S1 **Experimental parameters for precursor polymers (suffix –P).** a) propyl-containing polymers (Series 1), b) butyl-containing polymers (Series 2).
(DOCX)

Table S2 **Number-average molecular weight (M_n_) of SMAMP precursor polymers and polydispersity index (M_w_/M_n_) obtained by GPC (CHCl_3_, PMMA standards).**
(DOCX)

Text S1 **Supporting information text.**
(DOCX)
