# Supplementary figures and images for: Nature-Inspired Antimicrobial Polymers – Assessment of Their Potential for Biomedical Applications
Source: PLoS One. 2013 Sep 9;8(9):e73812. doi: 10.1371/journal.pone.0073812 (PMC3767731; doi:10.1371/journal.pone.0073812)

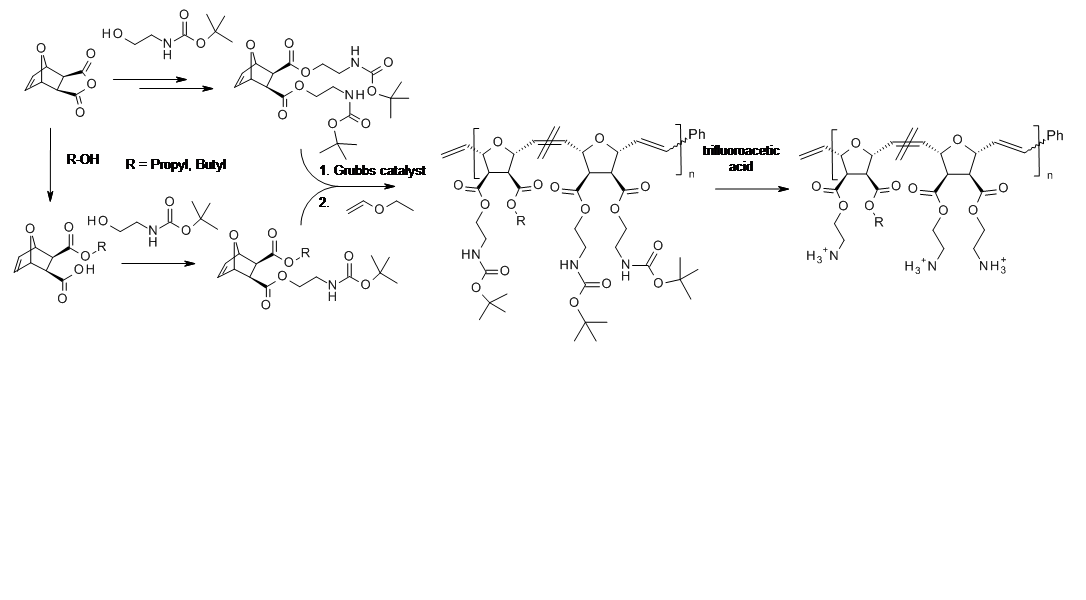

Supplement: Figure S1 — Copolymer synthesis. The monomers were obtained by ring-opening of oxonorbornene anhydride with the respective alcohol. The unreacted acid group was then further esterified. The monomers were mixed in the appropriate ratio (Table S1) and polymerized using Grubbs 3rd generation catalyst. After quenching the living polymerization with ethylvinyl ether, deprotection with trifluoroacetic acid yielded the desired SMAMP copolymers. (TIF) [file pone.0073812.s001.tif]

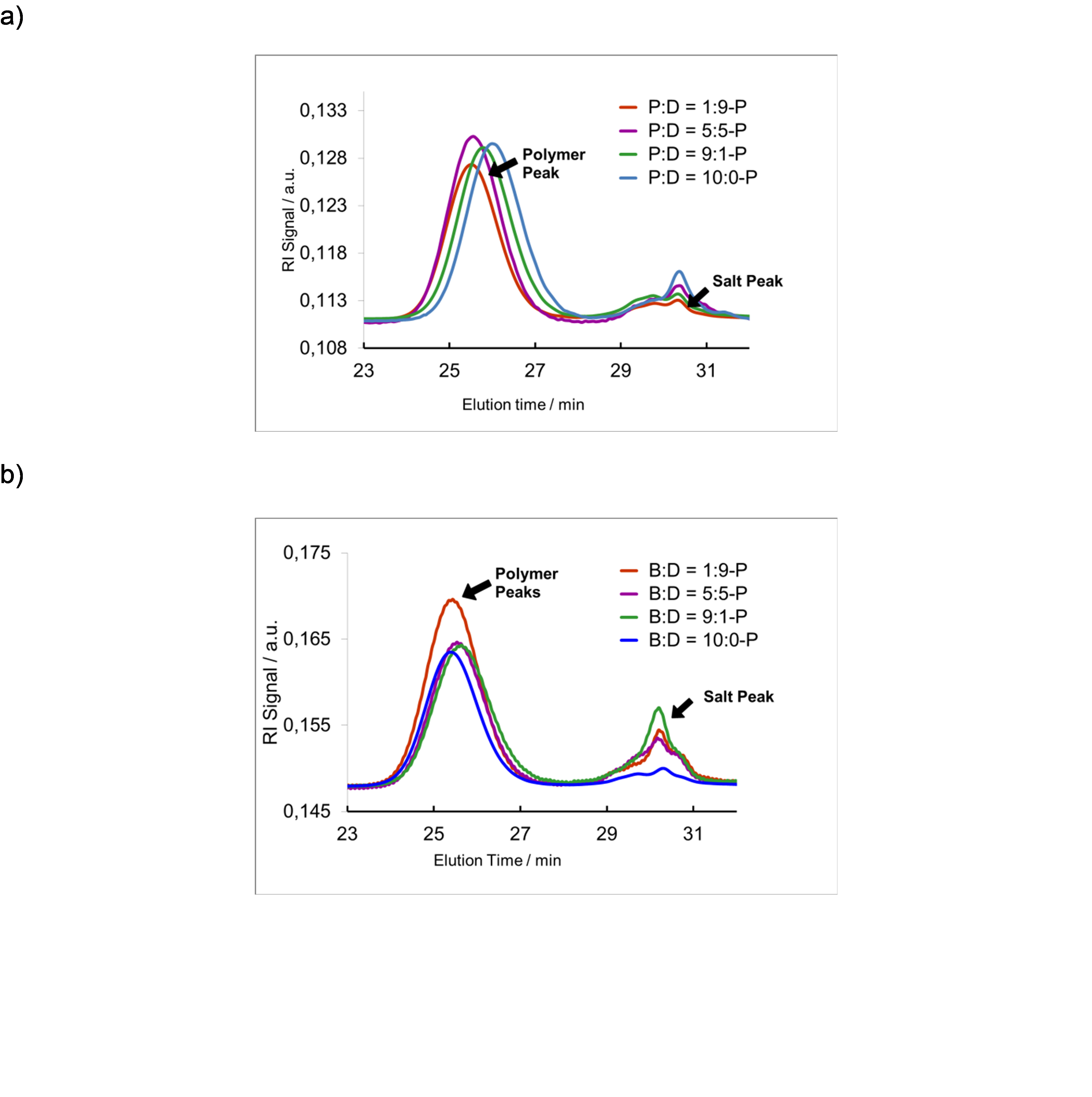

Supplement: Figure S2 — Overlay of GPC elugrams (refractive index detector signal (in arbitrary units) vs. elution time) of the precursor polymers (suffix –P). a) propyl-containing polymers (Series 1), b) butyl-containing polymers (Series 2). (TIF) [file pone.0073812.s002.tif]

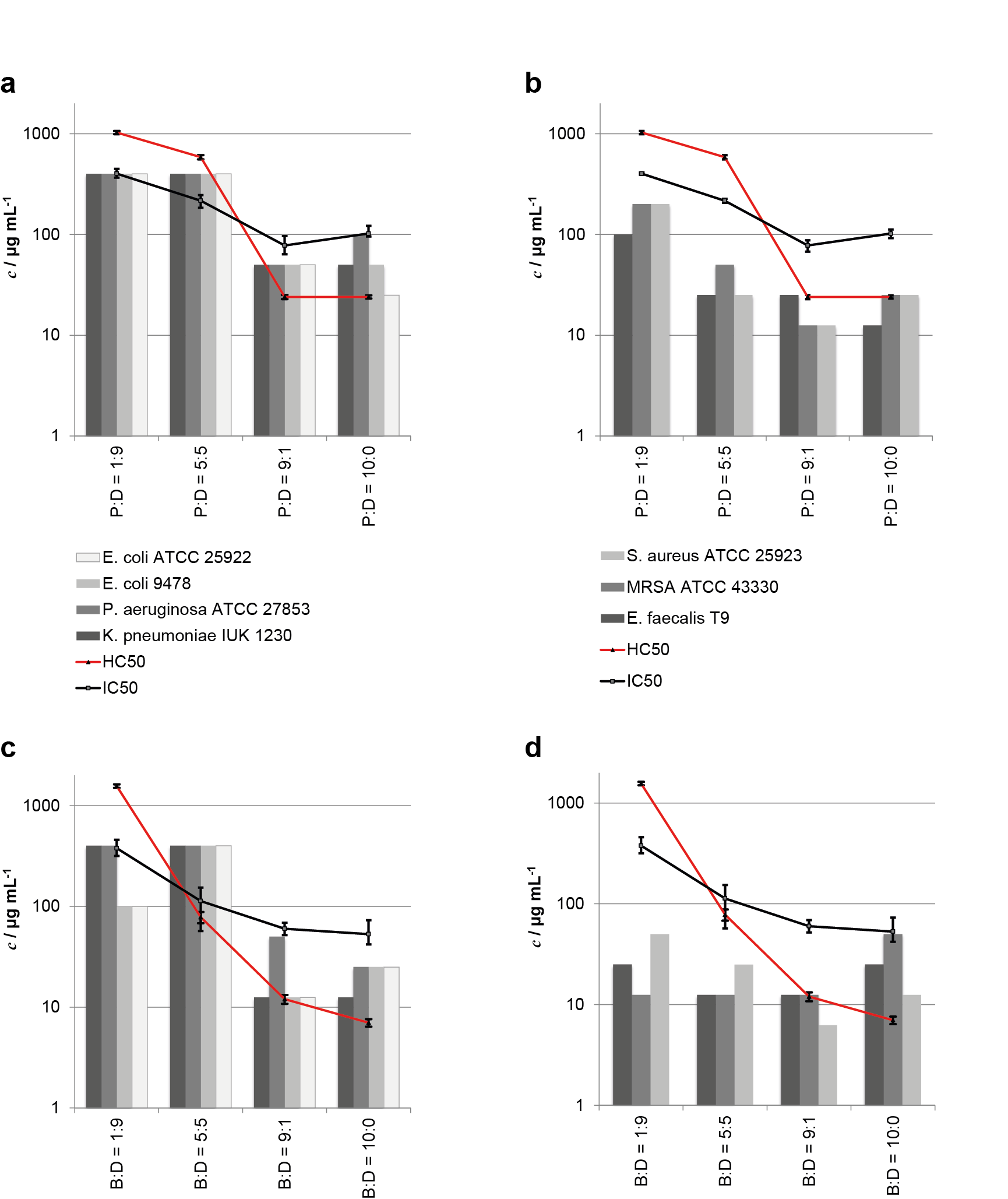

Supplement: Figure S3 — 1H-NMR spectra (250 MHz, CDCl3) of precursor polymers (suffix –P). a) propyl-containing polymers (Series 1), b) butyl-containing polymers (Series 2). (TIF) [file pone.0073812.s003.tif]

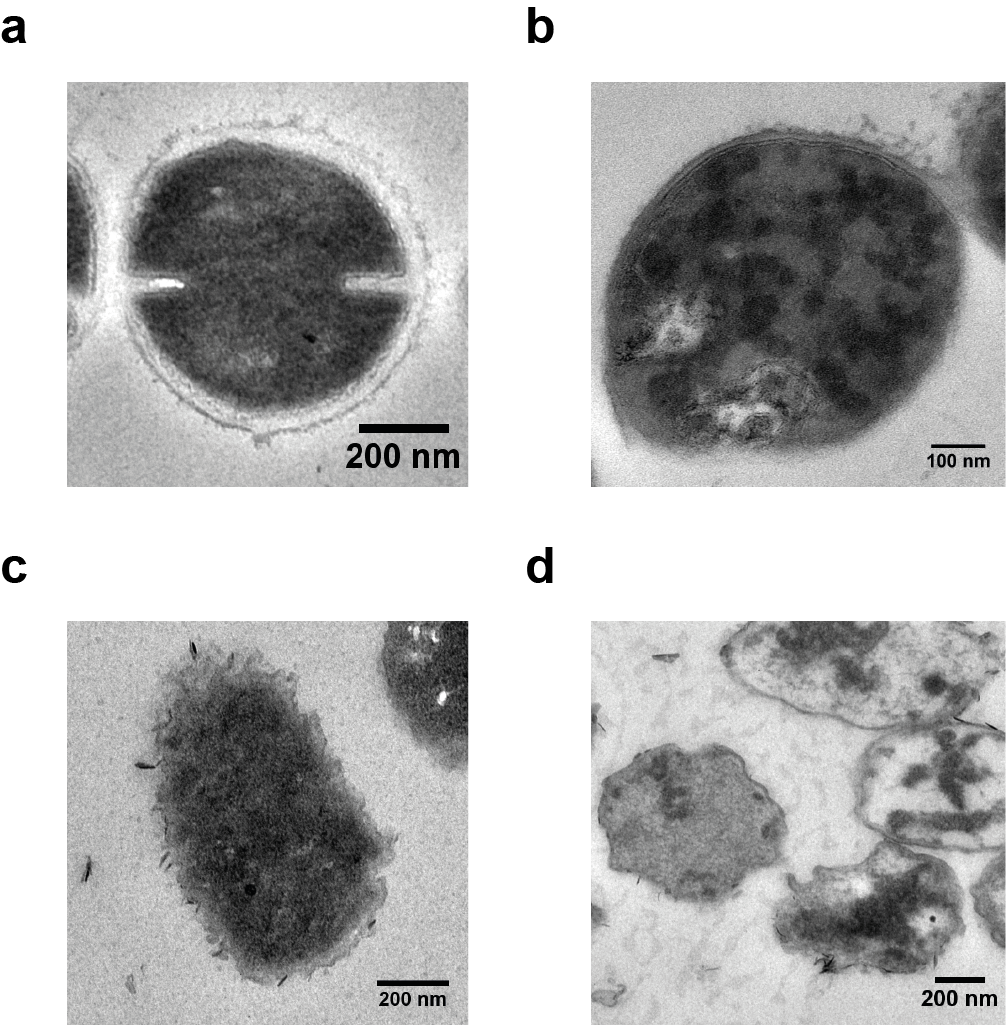

Supplement: Figure S4 — 1H-NMR spectra (250 MHz, CDCl3) of the SMAMP polymers. a) propyl-containing polymers (Series 1), b) butyl-containing polymers (Series 2). (TIF) [file pone.0073812.s004.tif]

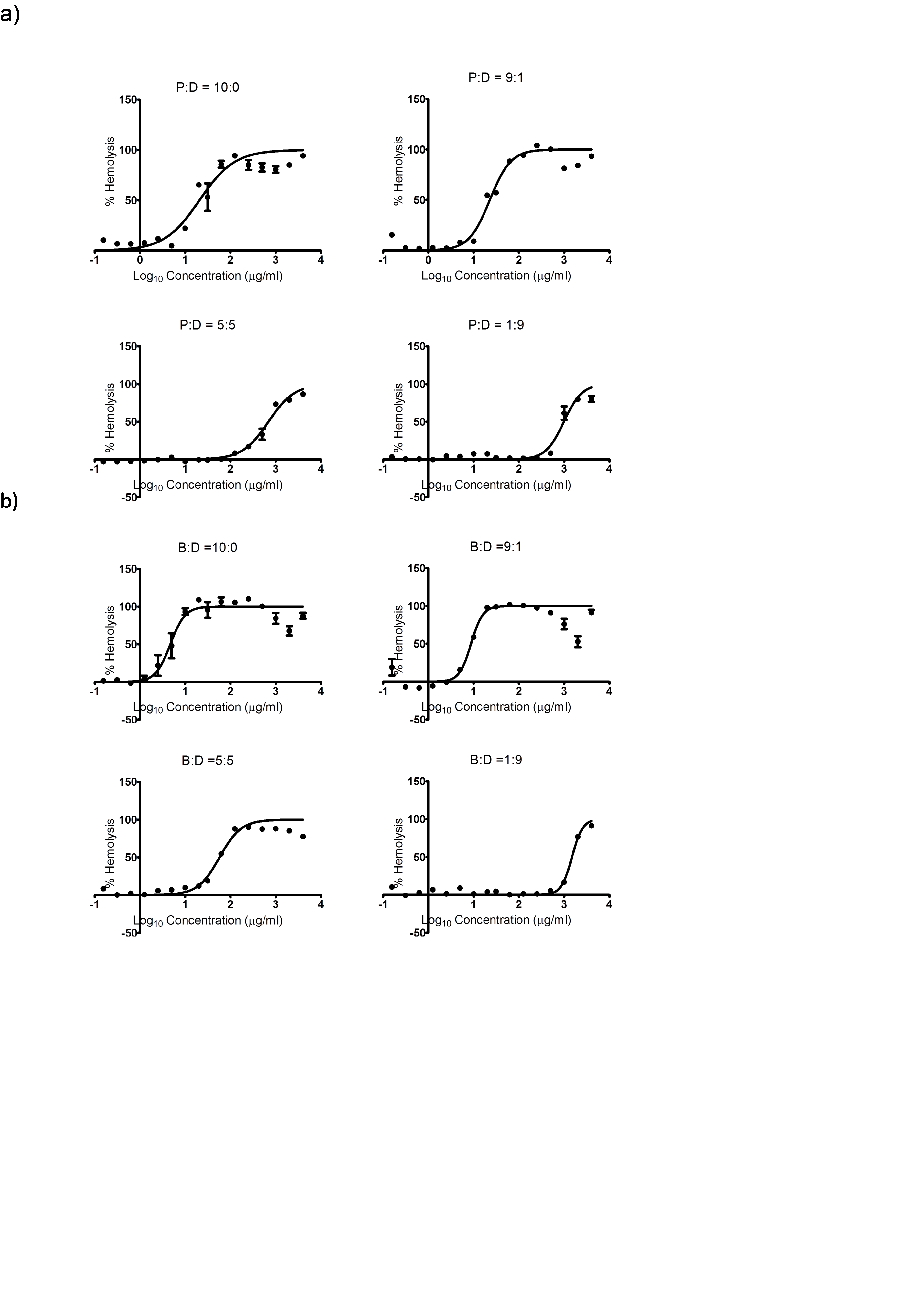

Supplement: Figure S5 — Results of the hemolysis assay. The percentage of hemolysis is plotted vs. log10 of SMAMP concentration, yielding the HC50 at the point of inflection; a) Series 1, b) Series 2. (TIF) [file pone.0073812.s005.tif]

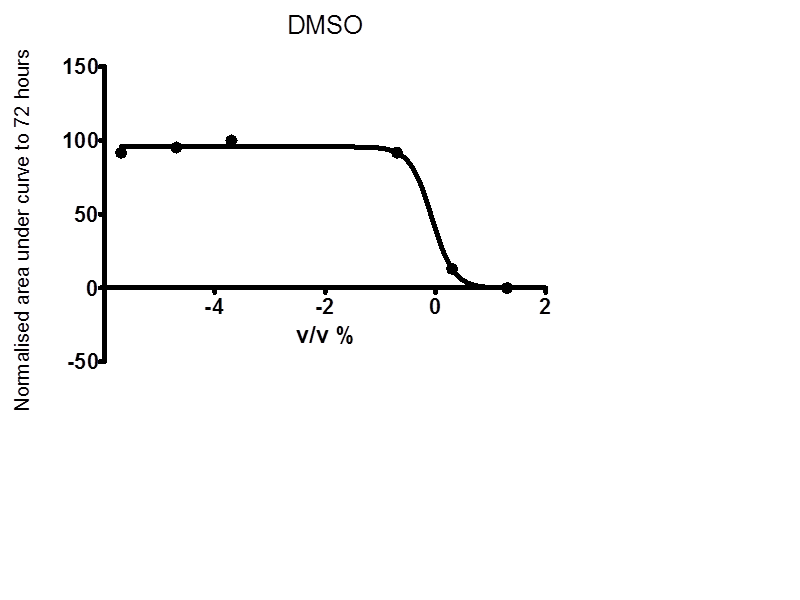

Supplement: Figure S6 — Sigmoidal curve fitted to the concentration dependent effect of DMSO on the proliferation of gingiva fibroblast cells over 72 hours (IC50 = 0.86 v/v %). (TIF) [file pone.0073812.s006.tif]

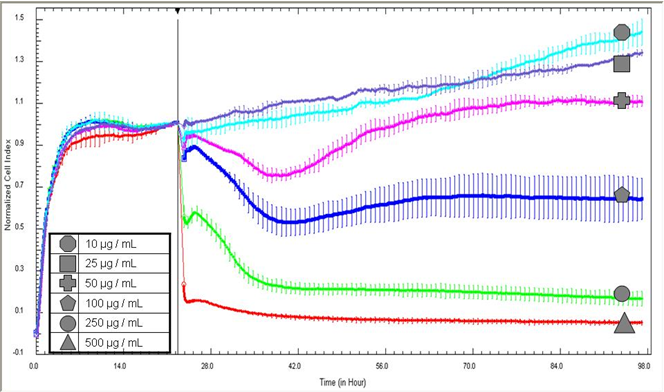

Supplement: Figure S7 — xCelligence plot showing the effect of SMAMP B:D = 1∶9 at various concentrations on gingiva fibroblast cell proliferation. (TIF) [file pone.0073812.s007.tif]

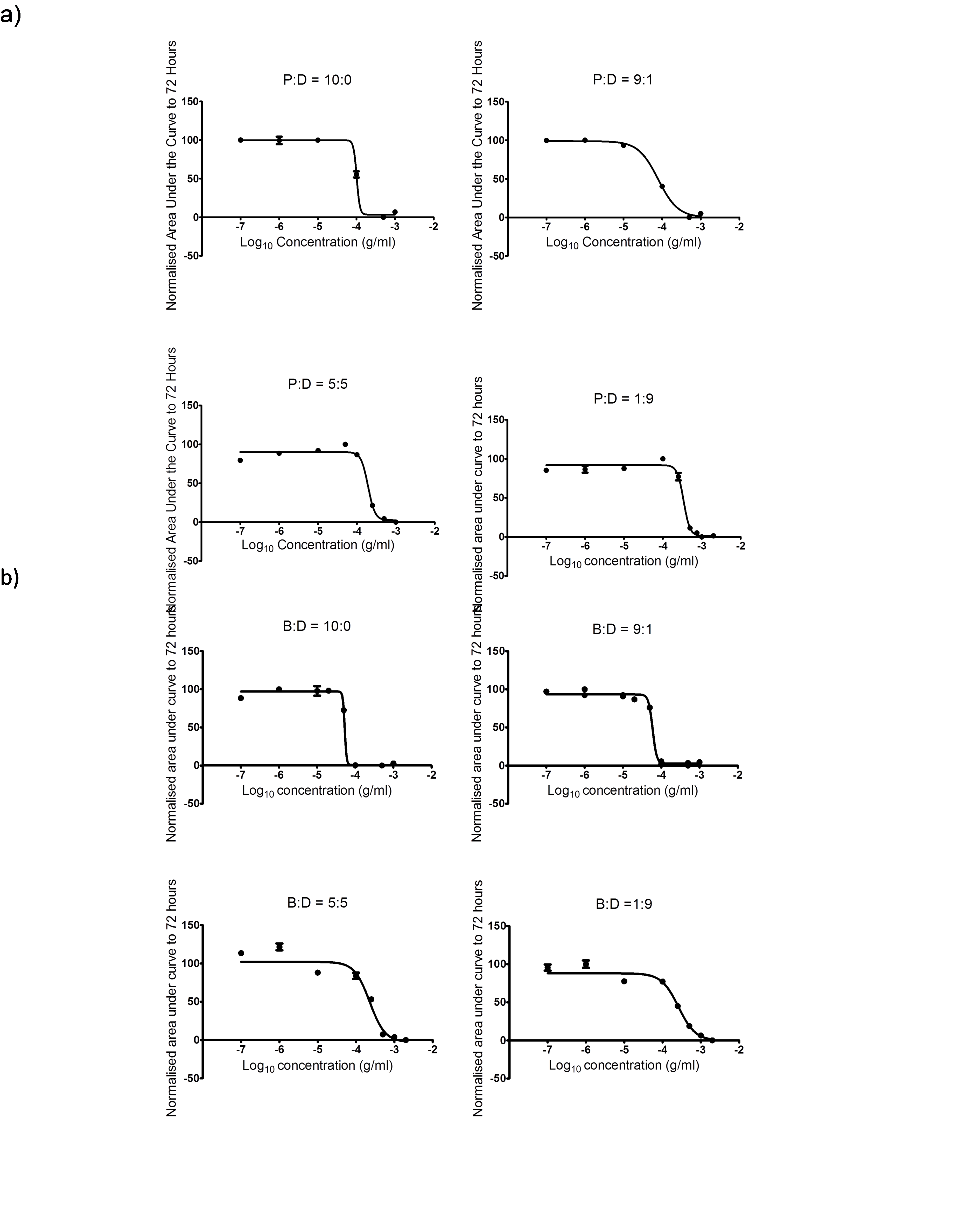

Supplement: Figure S8 — Results of the xCelligence assay. The normalized area under the curve is plotted vs. log10 of SMAMP concentration, yielding the IC50 at the point of inflection; a) Series 1, b) Series 2. (TIF) [file pone.0073812.s008.tif]
