# Supplementary material for: Nature-Inspired Antimicrobial Polymers – Assessment of Their Potential for Biomedical Applications
Source: PLoS One. 2013 Sep 9;8(9):e73812. doi: 10.1371/journal.pone.0073812 (PMC3767731; doi:10.1371/journal.pone.0073812)
Supplement: Table S1 — Experimental parameters for precursor polymers (suffix –P). a) propyl-containing polymers (Series 1), b) butyl-containing polymers (Series 2). (DOCX) [file pone.0073812.s009.docx]

Table S1: Experimental parameters for precursor polymers (suffix –P):

a) propyl-containing polymers (Series 1), b) butyl-containing polymers (Series 2)

a)

| **Series 1** | | | | | | | |
| --- | --- | --- | --- | --- | --- | --- | --- |
| **Sample** | **N*_Repeat units_*** | **n*_Propyl_***  (mmol) | ***m_Propyl_***  (mg) | **n*_Diamine_***  (mmol) | **m*_Diamine_***  (mg) | **n*_Catalyst_***  (mmol) | **m_Catalyst_**  (mg) |
| P:D = 10:0-P | 10 | 1.35 | 500.0 | 0 | 0 | 0.14 | 98.4 |
| P:D = 9:1-P | 10 | 1.21 | 446.5 | 0.14 | 65.8 | 0.14 | 98.4 |
| P:D = 5:5-P | 10 | 0.68 | 250.9 | 0.68 | 319.6 | 0.14 | 98.4 |
| P:D = 1:9-P | 10 | 0.14 | 51.7 | 1.21 | 568.7 | 0.14 | 98.4 |

b)

| **Series 2** | | | | | | | |
| --- | --- | --- | --- | --- | --- | --- | --- |
| **Sample** | **N*_Repeat units_*** | **n*_Butyl_***  (mmol) | ***m_Butyl_***  (mg) | **n*_Diamine_***  (mmol) | **m*_Diamine_***  (mg) | **n*_Catalyst_***  (mmol) | **m_Catalyst_**  (mg) |
| B:D = 10:0-P | 10 | 1.35 | 517.1 | 0 | 0 | 0.14 | 98.4 |
| B:D =9:1-P | 10 | 1.21 | 463.4 | 0.14 | 65.8 | 0.14 | 98.4 |
| B:D = 5:5-P | 10 | 0.68 | 260.4 | 0.68 | 319.6 | 0.14 | 98.4 |
| B:D = 1:9-P | 10 | 0.14 | 53.6 | 1.21 | 568.7 | 0.14 | 98.4 |
