# Supplementary material for: Nature-Inspired Antimicrobial Polymers – Assessment of Their Potential for Biomedical Applications
Source: PLoS One. 2013 Sep 9;8(9):e73812. doi: 10.1371/journal.pone.0073812 (PMC3767731; doi:10.1371/journal.pone.0073812)
Supplement: Table S2 — Number-average molecular weight (Mn) of SMAMP precursor polymers and polydispersity index (Mw/Mn) obtained by GPC (CHCl3, PMMA standards). (DOCX) [file pone.0073812.s010.docx]

Table S2: Number-average molecular weight (M_n_) of SMAMP precursor polymers and polydispersity index (M_w_/M_n_) obtained by GPC (CHCl_3_, PMMA standards)

| **Sample** | **M_n, Target_**  (g∙mol^-1^) | **GPC M_n,_**  (g∙mol^-1^) | **M_w_/M_n_** |
| --- | --- | --- | --- |
| P:D = 10:0-P | 3700 | 3300 | 1.1 |
| P:D = 9:1-P | 3780 | 3600 | 1.1 |
| P:D = 5:5-P | 4210 | 3700 | 1.1 |
| P:D = 1:9-P | 4580 | 4200 | 1.1 |
| B:D = 10:0-P | 3830 | 3600 | 1.1 |
| B:D =9:1-P | 3910 | 3500 | 1.1 |
| B:D = 5:5-P | 4280 | 3700 | 1.1 |
| B:D = 1:9-P | 4590 | 4000 | 1.1 |
