# Supplementary material for: Nature-Inspired Antimicrobial Polymers – Assessment of Their Potential for Biomedical Applications
Source: PLoS One. 2013 Sep 9;8(9):e73812. doi: 10.1371/journal.pone.0073812 (PMC3767731; doi:10.1371/journal.pone.0073812)
Supplement: Text S1 — Supporting information text. (DOCX) [file pone.0073812.s011.docx]

**Nature-inspired Antimicrobial Polymers – Assessment of Their Potential for Biomedical Applications**

Ali Al-Ahmad,^1,a^ Dougal Laird,^2,3,b^ Peng Zou,^2,4,b^ Pascal Tomakidi,^3,a^ Thorsten Steinberg,^3,a^ and Karen Lienkamp* ^2,4,a^

^1^ Operative Dentistry and Periodontology, University Medical Center of the Albert-Ludwigs-Universität, Freiburg, Germany

^2^ Freiburg Institute for Advanced Studies (FRIAS), Albert-Ludwigs-Universität, Freiburg, Germany

^3^ Oral Biotechnology, University Medical Center of the Albert-Ludwigs-Universität, Freiburg, Germany

^4^ Department of Microsystems Engineering (IMTEK), Albert-Ludwigs-Universität, Freiburg, Germany

* corresponding author: [lienkamp@imtek.uni-freiburg.de](mailto:lienkamp@imtek.uni-freiburg.de)

^a^ contributed equally

^b^ contributed equally

**Supporting Information:**

**Polymer Synthesis and Characterization:**

***SMAMP Precursor Polymers.***

All chemicals and solvents were obtained from Aldrich, Fluka or Acros, and used as received. The synthesis of the SMAMP monomers was described elsewhere, however the synthetic scheme is given in Figure S1.[1, 2] The protected precursors of the homopolymers and copolymers were synthesized as described below. The respective amounts of comonomers and Grubbs’ 3^rd^ generation catalyst for each polymerization are given in Table S1. Each was dissolved in 2 mL dry dichloromethane (DCM) under nitrogen. After stirring for 30 min at room temperature, the monomer solution was added in one shot to the catalyst solution. The reaction mixture was stirred at room temperature for a further 30 min, and the polymerization was stopped by adding ethylvinyl ether (1.0 mL, 750 mg, 10 mmol). The solution was then allowed to stir for 1 h. DCM was evaporated under reduced pressure. To get rid of the residual catalyst, the polymer was dissolved again in DCM (50 mL). Silica gel (1.0 g) was added and the mixture was stirred at room temperature for 2 h. The silica gel was then filtered out and the solvent was evaporated under reduced pressure. At last the polymer was dried under dynamic high vacuum.

All precursor polymers were characterized using gel permeation chromatography (GPC, PSS SDV columns, chloroform, poly(methylmethacrylate) (PMMA) standards, T = 30^o^C, flow rate 1.0 mL min^-1^). The resulting data is shown in Figure S3 and Table S2. All polymers had the targeted molecular weight and a low polydispersity index.

^1^H-NMR spectra (250 MHz, CDCl_3_) of all SMAMP precursors are shown in Figure S3. The chemical shifts of the protons in each repeat unit of copolymers matched the analogous protons in the homopolymers. The area ratio of 5-CH_2_ and 5’-CH_2_ peaks (4.0 – 4.3 ppm) to the peak of 6-CH_2_ (3.38 ppm) indicated that the ratio of repeat units in copolymers matched the molar ratio of the added comonomers. For simplicity, the ^1^H-NMR data for each repeat unit are given here separately.

Propyl repeat unit: ^1^H-NMR (250 MHz, CDCl_3_): 0.94 (t, 7’-CH_3_), 1.45 (s, 3 × 9-CH_3_), 1.68 (m, 6’-CH_2_), 3.13 (br m, 3-H & 3’-H), 3.37 (br m, 6-CH_2_), 4.01-4.24 (m, 5-CH_2_ & 5’-CH_2_), 4.73 (br m, 2-H & 2’-H, trans), 5.12 (br m, 2-H & 2’-H, cis), 5.42 (br, NH), 5.61 (br m, 1-H & 1’-H, cis), 5.92 (br m, 1-H & 1’-H, trans).

Butyl repeat unit: ^1^H-NMR (250 MHz, CDCl_3_): 0.94 (t, 8’-CH_3_), 1.28–1.51 (m, 7’-CH_2_ & 3 × 9-CH_3_), 1.62 (m, 6’-CH_2_), 3.13 (br m, 3-H & 3’-H), 3.37 (br m, 6-CH_2_), 4.01-4.24 (m, 5-CH_2_ & 5’-CH_2_), 4.73 (br m, 2-H & 2’-H, trans), 5.13 (br m, 2-H & 2’-H, cis), 5.41 (br, NH), 5.61 (br m, 1-H & 1’-H, cis), 5.91 (br m, 1-H & 1’-H, trans).

Diamine repeat unit: ^1^H-NMR (250 MHz, CDCl_3_): 1.45 (s, 6 × 9-CH_3_), 3.13 (br m, 2 × 3-H), 3.37 (br m, 2 × 6-CH_2_), 4.19 (br m, 2 × 5-CH_2_), 4.72 (m, 2-H, trans), 5.12 (br s, 2-H, cis), 5.46 (br, 2 × NH), 5.63 (br m, 1-H, cis), 5.91 (br m, 1-H, trans).

***SMAMP Polymers.***

120 mg of the respective precursor polymer were dissolved in DCM (4 mL). Trifluoroacetic acid (TFA, 4 mL, 6.0 g, 52 mmol) was added, and the solution was stirred at room temperature overnight. TFA was removed by azeotropic distillation under reduced pressure with DCM (2 × 10 mL) and methanol (2 × 5 mL). After drying, the polymer was dissolved in a methanol-water mixture (MeOH : H_2_O = 1 : 4, 25mL) and dialyzed against Milli-Q water until the conductivity of the water stay at 0.1 μΩ for at least 24 h (total dialysis time 6-8 days). Longer hydrolysis times are not recommended as the polymer may be involved in water-catalyzed side reactions. In the last step, the hydrolyzed polymer was freeze-dried to obtain the SMAMP as yellow-brown powder.

The ^1^H-NMR analysis (Figure S4) showed a complete deprotection of the Boc-protected amine groups. Because of the inevitable adsorption of water, which shows a broad peak from 3.0 to 3.6 ppm, a calculation for the area ratio of 5-CH_2_ and 5’-CH_2_ peaks to the 6-CH_2_ peak is not possible. However, the increasing relative intensity of the NH_3_^+^ peak along with the increasing relative amount of diamine indicated that the composition of SMAMPs matched the composition of the precursors.

Propyl repeat unit: ^1^H-NMR (250 MHz, DMSO-d_6_): 0.86 (br m, 7’-CH_3_), 1.23 (m, NH_2_), 1.55 (br m, 6’-CH_2_), 3.05 (br m, 3-H & 3’-H), 3.36 (br m, 6-CH_2_), 3.89-4.37 (m, 5-CH_2_ & 5’-CH_2_), 4.43-4.70 (br m, 2-H & 2’-H, trans), 4.77-5.13 (br m, 2-H & 2’-H, cis), 5.44-5.73 (br m, 1-H & 1’-H, cis), 5.76-5.90 (br m, 1-H & 1’-H, trans), 8.14 (br s, NH_3_^+^).

Butyl repeat unit: ^1^H-NMR (250 MHz, DMSO-d_6_): 0.87 (br m, 8’-CH_3_), 1.05-1.38 (m, 7’-CH_2_ & NH_2_), 1.52 (br m, 6’-CH_2_), 3.05 (br m, 3-H & 3’-H), 3.34 (br m, 6-CH_2_), 3.87-4.37 (m, 5-CH_2_ & 5’-CH_2_), 4.41-4.70 (br m, 2-H & 2’-H, trans), 4.75-5.11 (br m, 2-H & 2’-H, cis), 5.44-5.72 (br m, 1-H & 1’-H, cis), 5.74-5.98 (br m, 1-H & 1’-H, trans), 8.14 (br s, NH_3_^+^).

Diamine repeat unit: ^1^H-NMR (250 MHz, DMSO-d_6_): 1.23 (m, 2 × NH_2_), 3.09 (br m, 2 × 3-H), 3.49 (br m, 2 × 6-CH_2_), 4.22 (m, 2 × 5-CH_2_), 4.52-4.74 (br m, 2 × 2-H, trans), 4.85-5.12 (br m, 2 × 2-H, cis), 5.43-5.74 (br m, 2 × 1-H, cis), 5.76-5.94 (br m, 2 × 1-H, trans), 8.14 (br s, 2 × NH_3_^+^).

**Microbiological Studies:**

***Bacterial Strains.***

Seven different bacterial strains belonging to 5 species were used. *Staphylococcus aureus* ATCC 25923, *Staphylococcus aureus* (MRSA) ATCC 43300 and *Enterococcus faecalis* T9 are Gram-positive species, whereas *Escherichia coli* ATCC 25922, *Escherichia coli* 9478, *Pseudomonas aeruginosa* ATCC 27853 and *Klebsiella pneumoniae* IUK 1230 have a Gram-negative cell wall. *E. faecalis* T9, *E. coli* 9478 and *K. pneumonia* IUK 1230 were kindly provided by the Division of Infectious Diseases, the Institute of Medical Microbiology and Hygiene and the Department of Environmental Health Sciences of the Albert-Ludwigs-University, Freiburg, respectively. Long-term storage of these microorganisms was conducted at -80°C in basic growth medium containing 15% (v/v) glycerol as described by Jones *et al.*. [3]

***Determination of the Minimum Inhibitory Concentration (MIC and MIC_90_):***

An overnight culture of each bacterial strain was prepared. All strains were tested at 10^6^ colony forming units (CFU) mL^-1^ in Mueller-Hinton Broth (MHB). Using a multi-channel pipette, a 96-well microtiterplate was inoculated with the appropriate volumes of bacterial cultures previously prepared in MHB. Stock solutions of the SMAMP polymers were prepared in dimethyl sulfoxide (DMSO, Sigma, Steinheim, Germany). All polymers were tested in MHB containing residuals of DMSO in a concentration series ranging from 400 µg mL^-1^ to 6.25 µg mL^-1^. The total volume in each well was 150 µL. In parallel, a dilution series of DMSO was also tested to exclude possible effects of DMSO residuals. Inoculated wells with MHB which contained neither polymers nor DMSO served as positive control for bacterial growth. Sterile MHB was taken as blank to exclude any contamination. The plates were incubated for 18 h at 37°C in an aerobic atmosphere with 5% CO_2_. After incubation, the optical density (OD) at 600 nm was determined using a Tecan Infinite 200 plate-reader (Tecan, Crailsheim, Germany). All tests for each strain were carried out at least in duplicate. The MIC was defined as the lowest concentration of each polymer that was able to inhibit bacterial growth, and is reported with the percentage of bacterial growth at that concentration. The MIC_90_ was defined as the concentration at which at least 90% growth reduction was observed. Any inhibition effects caused by DMSO were accounted for by considering the growth of the DMSO series conducted in parallel.

***Determination of the Minimum Bactericidal Concentration (MBC):***

After incubation of the microtiter plates used for testing the MIC, 100 µl from each well of the concentration series were plated on Columbia blood agar (CBA) plates. The agar plates were then incubated for three days at 37°C in an aerobic atmosphere with 5% CO_2_. The colony forming units (CFU) were counted using the Gel Doc EQ Universal Hood (Bio-Rad Life Science Group, Hercules, USA). The MBC is defined as the concentration where a three log killing of bacterial growth (=99.9%) is observed compared to a control.

***Transmission Electron Microscopy (TEM):***

Bacterial cells were fixed overnight in phosphate-buffered solution containing 4% paraformaldehyde and 2.5% glutaraldehyde (Polyscience, Eppelheim, Germany). Afterwards, the fixed bacterial cells were washed using 100 mM phosphate buffer. A post-fixation was conducted in 6.86% sucrose buffer containing 1% osmium tetroxide at room temperature in the dark. The samples were washed with 100 mM phosphate buffer, contrasted in 1% uranyl acetate for 60 min, and dehydrated in an ascending ethanol series (50-100%). After 1 h incubation in a propylene oxide-Durcopan mixture (Durcopan ACM, Sigma-Aldrich, 1:1), the samples were incubated in Durcopan and flat-embedded overnight. After sample hardening for two days at 55°C, sections were cut at a thickness of 55 nm using an ultramicrotome (Leica EM UC 6, Wetzlar, Germany). The sections were collected on Formvar-coated slot copper grids. They were then contrasted with 1% uranyl acetate for 30 min, washed with double distilled water, and contrasted with Reynolds' lead citrate for three min. The ultrathin sections were visualized using a Philips CM 100 electron microscope (Philips, Eindhoven, Netherlands). Image analysis was conducted using instrument-specific software (Gatan GmbH, München, Germany).

**Cell Compatibility Studies**

***Ethics statement:***

All experiments involving human material have been carried out in accordance to the guidelines of the World Medical Association Declaration of Helsinki. Primary human gingival fibroblasts (GF) were obtained using the explant technique.[4] For tissue harvest, informed written consent was obtained from the patients, and the explant protocol was approved by the institutional ethics committee (Ethik-Kommission der Albert-Ludwigs-Universität Freiburg, Germany, vote 411/08_121010). Human red blood cells (erythrocytes) were obtained from the full blood of volunteers who have given their informed written consent; this was approved by the institutional ethics committee (Ethik-Kommission der Albert-Ludwigs-Universität Freiburg, Germany).

***Haemolysis Assays:***

Haemolysis experiments were performed according to the method described by Mowery *et. al.* with minor modifications.[5] In short, fresh blood was drawn from a human volunteer on the day of the experiment and washed with Tris-buffered saline (TBS, pH 7.2, 0.01 M Tris-HCl, 0.155 M NaCl) by adding 0.5 mL blood to 10 mL TBS in a 15 mL Greiner tube, shaking well by hand, and then centrifuging at 1000 g for 5 minutes. The supernatant above the concentrated red blood cells (RBCs) was discarded and a further 10 mL TBS added. This procedure was repeated 3 times to yield plasma free RBCs, which were diluted for experimental use to 2% v/v RBCs in TBS (200 µL RBCs filled up to 10 mL with TBS).

The SMAMP samples were prepared as stock solutions in dimethyl sulfoxide (DMSO) at 80 mg mL^-1^ and diluted with TBS to give two working solutions at 8000 µg mL^-1^ and 40 µg mL^-1^. Serial dilutions were made in a 96-well plate (Bio-One, Cellstar, Greiner) so that concentrations were stepwise halved 8 times to give a range from 4000 µg mL^-1^ to 31.25 µg mL^-1^ and a range from 40 mµ mL^-1^ to 0.3125 µg mL^-1^ in 50 µL TBS. 10 µL of a 1 mg mL^-1^ stock solution of the haemolytic compound melittin was added to 40 µL TBS as a positive control, 50 µL TBS as control (blank) and 100 µL TBS as background control. Finally, 50 µL of the 2% RBC solution was added to each well containing 50 µL sample or control. The plate was shaken gently for 1 minute then incubated for 1 hour at 37°C. Thereafter, the plate was centrifuged at 1000 g for 5 minutes, subsequent to which 80 µL of the supernatant was pipetted into a new 96 well plate and the optical density (OD) was measured at 414 nm on a UV-vis plate reader (Tecan infinite M200).

For statistical analysis, blank OD_414_ values were subtracted from both sample OD_414_ and positive control OD_414_ values. OD_414_ was normalised to 100 % with the lowest OD_414_ assigned to 0 % haemolysis, and the melittin positive control assigned to 100 % haemolysis. The data was plotted as OD_414_ vs. log_10_ of the concentration using the graphing software Graphpad Prism. A sigmoidal curve was fitted to the data by the software to yield an HC_50_ value and 95% confidence intervals. The curves of percentage hemolysis vs. log_10_ of concentration for all SMAMPs are shown in Figure S5. The HC_50_ values reported are the average values derived from 3 independent hemolysis experiments, and error bars represent the Standard Error of the Mean (SEM).

***xCELLigence Cell Compatibility Assay:***

*Method:* Primary human gingival fibroblasts (GF) were obtained using the explant technique.[3] Cultures were maintained for routine cell culture in Dulbecco's Modified Eagle Medium (DMEM, PAA, Pasching, Austria) containing 10% fetal calf serum (Seromed, Biochrom, Berlin, Germany), Glutamax (5 mL in 500 mL medium, 100x, Gibco by Life Technologies) and 50µg/ml kanamycin (Roche Diagnostics, Mannheim, Germany). GFs were cultured in passages 8-12 and comparable passages were used for seeding in 16-well E-plates. To exclude cell-senescence caused alterations of cell morphology and expression of tissue-specific biomarkers, periodontal cells were tested previously on consistent expression of respective biomarkers and stable morphology over time.

xCELLigence experiments were performed in accordance with the manufacturer’s (Roche) recommendations. 100 µL pre-warmed (37°C) DMEM medium (PAA, Pasching, Austria) containing 10 % fetal calf serum (Seromed, Biochrom, Berlin, Germany), Glutamax (5 mL in 500 mL medium, 100x, Gibco by Life Technologies), and 50 µg/ml Kanamycin (Roche Diagnostic, Mannheim, Germany) was pipetted into all wells of a 16 well E-plate (Roche) for impedance spectroscopy measurements. The E-plate was then inserted into the xCELLigence instrument and allowed to equilibrate for 30 minutes, before a background measurement of the impedance was performed. Thereafter, 100 µL of a suspension of human gingiva fibroblast (GF) cells in DMEM medium was added to each of the wells to give 5 x 10^3^ cells per well (1). These seeded cells were allowed to attach and proliferate in a cell incubator overnight.

SMAMP stock solutions were prepared as 80 mg mL^-1^ in DMSO and diluted with pre-warmed DMEM medium to yield concentrations from 1.0 mg mL^-1^ to 0.1 ng mL^-1^ (2). Approximately 18 hours after initial cell seeding, 100 µL medium was carefully removed from above the cultivated cells (1) and 100 µL of the SMAMP/medium solution (2) was added so that an overall concentration range of 1 mg mL^-1^ to 0.1 ng mL^-1^ resulted. This was done in duplicate. Subsequent replicate experiments were focused on the concentration range at the point of inflexion of the IC_50_ curve obtained. 20% DMSO was added as a positive control, while the exchange of 100 µL of medium from the well with fresh DMEM medium was carried out as a negative control. Additionally, an experiment involving the combination of DMSO with DMEM medium at the same volume/volume ratio as that present in the SMAMP samples was undertaken to confirm that observed cytotoxic effects were not due to the effects of DMSO as shown in Figure S6. A DMSO volume percent IC_50_ value of 0.86% (687 µg/mL) resulted, corresponding to the volume percent composition in a SMAMP solution. All IC_50_ values for the SMAMPs were lower than this value (highest 400 µg/mL), indicating that activity was due to the SMAMP and not DMSO alone.

Following the addition of the SMAMP solution to the GF cells, the impedance measurement was allowed to run for at least a further 72 hours. The impedance signal was recorded as a function of time. An example of the resulting plot is shown in Figure S7. The data was extracted from the resulting plot by first applying an xCELLigence software (RCTA Software) generated normalization at the time point at which the E-plate was removed for the addition of the 100 µl SMAMP in medium solution (normalized value = measured impedance/impedance at the time of normalization, yielding the cell index (CI)). Next, the area under to the curve was extracted using the RCTA software from the point of normalization to 72 hours thereafter. All data was entered into the bio-statistical software GraphPad Prism, wherein the area under the curve normalized to 100% (highest value = 100 %, lowest value = 0 %) was plotted against log_10_ of the concentration. Thereafter, the data was processed according to the “sigmoidal dose-response” (variable slope nonlinear regression curve fit for an inhibitor), from which the IC_50_ was obtained. The software generated a standard error value for the data points based on the fitted sigmoidal curve and the variability of the points around that curve. From this standard error value, a 95% confidence interval for the IC_50_­ value was calculated and given as error bars in Fig. 3. The IC_50_ curves for all samples are shown in Figure S8.

1 Lienkamp K, Madkour A E, Musante A, Nelson C F, Nusslein K et al. (2008). J Am Chem Soc 130: 9836-9843

2 Lienkamp K, Madkour A E, Kumar K N, Nusslein, Tew G N (2009) Chem Eur J 15: 11715-11722

3 Jones D, Pell P A, Sneath P H A, in *Maintenance of Microorganisms and Cultured Cells: A Manual of Laboratory Methods*, eds. B. E. Kirsop and A. E. Doyle, Academic Press, London, 2nd edn., 1991, pp. 45-50.

4 Tomakidi P, Breitkreutz D, Fusenig N E, Zoller J, Kohl A et al. (1998), *Cell Tissue Res* 292: 355-366

5 Mowery B P, Lindner A H, Weisblum B, Stahl S S, Gellman S H (2009), J Am Chem Soc 131: 9735-9745
